# Cancer-related fatigue among adult patients living with cancer in Ethiopia: A systematic review and meta-analysis

**DOI:** 10.1093/noajnl/vdag105

**Published:** 2026-04-17

**Authors:** Yilkal Abebaw Wassie, Berhan Tekeba, Masresha Asmare Techane, Chalachew Adugna Wubneh, Bezawit Dereje Tilahun, Tadele Mesfin Demelash, Fraol Zeleke Desta, Cherugeta Kebede Asfaw, Elsabeth Alemayehu Haile, Simon Zemenfes Hailu, Frehiwot Tekie Woldemichael, Yohana Moges Mehari, Henok Dessie Wubneh, Mulat Alemu Beshada, Ermiyas Meskelu Adugna, Leul Alemgena Yohannes, Tesfaye Birhanu Abebe, Tadesse Tarik Tamir

**Affiliations:** Department of Medical Nursing, School of Nursing, College of Medicine and Health Sciences, University of Gondar, Gondar, Ethiopia; Department of Pediatrics and Child Health Nursing, School of Nursing, College of Medicine and Health Sciences, University of Gondar, Gondar, Ethiopia; Department of Pediatrics and Child Health Nursing, School of Nursing, College of Medicine and Health Sciences, University of Gondar, Gondar, Ethiopia; Department of Pediatrics and Child Health Nursing, School of Nursing, College of Medicine and Health Sciences, University of Gondar, Gondar, Ethiopia; School of Medicine, College of Medicine and Health Sciences, University of Gondar, Gondar, Ethiopia; School of Medicine, Asrat Woldeyes Health Science Campus, Debre Berhan University, Debre Berhan, Ethiopia; School of Medicine, College of Medicine and Health Sciences, Salale University, Fitche, Ethiopia; School of Medicine, College of Medicine and Health Sciences, Salale University, Fitche, Ethiopia; School of Medicine, College of Medicine and Health Sciences, Mekelle University, Mekelle, Ethiopia; School of Medicine, College of Medicine and Health Sciences, Mekelle University, Mekelle, Ethiopia; School of Medicine, College of Medicine and Health Sciences, Mekelle University, Mekelle, Ethiopia; School of Medicine, College of Medicine and Health Sciences, Mekelle University, Mekelle, Ethiopia; School of Medicine, College of Medicine and Health Sciences, University of Gondar, Gondar, Ethiopia; School of Medicine, College of Medicine and Health Sciences, Salale University, Fitche, Ethiopia; School of Medicine, College of Medicine and Health Sciences, Salale University, Fitche, Ethiopia; School of Medicine, College of Medicine and Health Sciences, Salale University, Fitche, Ethiopia; School of Medicine, College of Medicine and Health Sciences, Salale University, Fitche, Ethiopia; Department of Pediatrics and Child Health Nursing, School of Nursing, College of Medicine and Health Sciences, University of Gondar, Gondar, Ethiopia

## Abstract

**Background:**

Cancer-related fatigue (CRF) is one of the most common and distressing side effects of cancer and its treatment. Its clinical impact contributes to poorer health outcomes, reduced quality of life, and higher healthcare costs. Although several primary studies have been conducted in Ethiopia, no systematic review has yet synthesized their findings on CRF.

**Methods:**

A comprehensive systematic search was conducted across major databases, including HINARI, Science Direct, Embase, Thesis Bank, PubMed/MEDLINE, Google Scholar, and Research Gate. Following a rigorous screening based on predefined inclusion criteria, 7 studies were included. Data extraction and quantitative synthesis were performed using STATA software. Statistical heterogeneity was assessed with Cochran’s *Q* and the *I*^2^ statistic, while publication bias was assessed via Egger’s regression test.

**Results:**

This systematic review and meta-analysis included 7 studies comprising 2047 participants, with a pooled prevalence of 73.04% (95% CI: 69.00-77.08%). The prevalence was highest in the Hawassa region (77.40%) and among studies conducted before 2020 (73.34%). Cancer-related fatigue was significantly associated with late-stage disease presentation, anemia, depression, and anxiety.

**Conclusion and Recommendations:**

Cancer-related fatigue is a significant public health issue in Ethiopia, with a high prevalence, especially in the Hawassa region. Late-stage cancer, anemia, depression, and anxiety were strong predictors of CRF. Routine screening for fatigue and psychological comorbidities should be integrated into oncology care to improve patient outcomes.

Key Points

- CRF is a very common and distressing symptom experienced by a large majority of adult cancer patients in Ethiopia.
- The findings identify a range of factors that are statistically and clinically associated with a higher likelihood of experiencing CRF.
- The findings suggest that CRF should be considered a “vital sign” and that all cancer patients in Ethiopia should be routinely screened for fatigue.

Importance of the Study

The findings underscore that CRF is not just a minor side effect but a major, widespread problem that significantly impacts patients’ quality of life. This can prompt a change in clinical practice, urging healthcare providers to move beyond a focus solely on treating the cancer itself and to actively screen for and manage CRF. By conducting a meta-analysis, the study can more definitively identify the most significant risk factors for CRF in the Ethiopian population. This goes beyond what individual studies can achieve, as it increases statistical power and allows for a more reliable understanding of the drivers of fatigue.

Cancer remains the second leading cause of global mortality and represents an intensifying public health crisis. Global cancer incidence is projected to rise from 12.7 million cases in 2008 to 21 million by 2030.^1^^,^^2^ This burden disproportionately affects the Globe; specifically, regions across Africa and the Americas now account for over 60% of annual cases. Sub-Saharan Africa faces a particularly steep increase, with cancer-related morbidity and mortality expected to rise by approximately 70%, driven by an aging population and rapid demographic growth projected to reach 60% by 2030.^3–5^ In Ethiopia alone, this trend is reflected in hospital records that have reached approximately 150 000 new cancer diagnoses annually, underscoring the urgent need for to strengthen oncologic care infrastructure.^6^

Cancer-related fatigue (CRF) stands as the most pervasive side effect of oncological treatment, surpassed only by pain in its global burden. It is characterized by a persistent, subjective sense of physical, emotional, and cognitive exhaustion that is not relieved by rest.^7^ Unlike general tiredness, CRF is a debilitating condition that fundamentally obstructs daily functioning and diminishes a patient’s quality of life.^8^ The clinical implications of significant CRF extend beyond the individual experience, frequently resulting in a deterioration of overall health status, prolonged hospitalizations, and a heightened reliance on ambulatory services, all of which drive a substantial increase in long-term healthcare expenditures.^9^

The reported prevalence of CRF exhibits significant variability, typically affecting between 25% and 99% of patients undergoing chemotherapy, with roughly one-third of survivors facing persistent symptoms.^10–12^ Global meta-analyses reveal diverse pooled prevalence estimates ranging from 26.9% in the Netherlands to 49% in the United States and 52% in China.^13–15^ These variations are largely driven by clinical context: prevalence ranges from 26.2% in gynecological cancers to 56.3% in mixed-type cohorts, peaking at 60.6% in advanced-stage disease. Furthermore, fatigue levels are influenced by treatment phases; they remain high during active treatment (62%) compared to mixed treatment phases (51%).^16^

A multimodal strategy is necessary for the effective therapy of cancer-related fatigue (CRF), since rest is not enough to stop the cycle of functional decline.^17^ The most effective way to reverse muscle atrophy and increase metabolic efficiency is still through physical exercise, particularly a combination of resistance training and aerobic activity. In addition, cognitive behavioral therapy (CBT) teaches activity pacing to avoid overexertion and enhances sleep hygiene to address the fatigue-distress cycle.^18–20^ Furthermore, mind-body therapies like yoga and tai chi provide dual advantages by lowering systemic inflammation and easing psychological stress and comorbidities like sleeplessness and depres‑sion that frequently make fatigue worse.^21–23^

CRF remains a formidable public health challenge that severely compromises a patient’s occupational and social functioning.^24^^,^^25^ While epidemiological insights are vital for refining cancer control strategies, there is a significant research gap regarding the Ethiopian context. Therefore, this study aims to provide the first systematic estimate of the pooled prevalence and associated risk factors of CRF among adult cancer patients in Ethiopia to inform more targeted clinical interventions.

## Research Questions About the Pooled Prevalence and Associated Factors of Cancer-Related Fatigue Among Adult Patients Living With Cancer

What is the pooled prevalence of cancer-related fatigue among adult cancer patients?
What are the pooled factors associated with cancer-related fatigue among adult cancer patients?

## Methods

### Study Protocol

This systematic review and meta-analysis were conducted in accordance with a publicly registered protocol available in PROSPERO (registration number CRD420250632584). To ensure methodological rigor and transparency, the protocol was developed prior to data extraction and analysis. Furthermore, the review meticulously follows the guidelines set forth by the Preferred Reporting Items for Systematic Reviews and Meta-Analyses (PRISMA) criteria^26^ (Supplementary Table S2). This ensures comprehensive reporting across all stages of the research, from literature identification and study selection to data extraction and synthesizing findings.

### Search Strategy

This systematic review and meta-analysis were undertaken to ascertain the prevalence and associated factors of cancer-related fatigue among adult patients living with cancer in Ethiopia. A comprehensive literature search was conducted across different databases, including HINARI, Science Direct, Embase, Thesis Bank, PubMed/MEDLINE, Google Scholar, and Research Gate, to identify relevant investigations. Additionally, the reference lists of included articles were manually screened for more pertinent studies.

The search was performed between October 9, 2024, and February 9, 2025, and included all online-available publications up to the final search date. The search terms used included “cancer-related fatigue,” “occurrence,” “cancer,” “chemotherapy,” “malignancy,” “prevalence,” “associated factors,” “predictors,” “determinant,” and “Ethiopia.” To identify suitable articles, these phrases were combined with “AND” and “OR.” Furthermore, we performed an attempt to email the primary or corresponding authors to get any information that was missing after the data was extracted from the papers.

### Study Selection

To identify all potentially relevant publications, comprehensive searches of multiple electronic databases were commenced. Once identified, duplicate publications were meticulously removed, and the remaining articles were imported into EndNote version 20 for efficient management. Three independent reviewers (Y.A.W., B.T., and T.T.T.) initially screened the titles and abstracts to determine their relevance to the research objectives. Following this preliminary review, the same investigators independently appraised the full-text papers according to the predetermined inclusion criteria. Any disagreements that arose during this crucial phase were resolved through discussion before the data extraction and analysis.

### Eligibility Criteria

#### Inclusion criteria

The prevalence of cancer-related fatigue in patients with any cancer disease who are admitted to hospital wards or visiting outpatient clinics is the subject of observational studies (cohort, cross-sectional, and case-control studies), reported as original articles, theses, and abstracts from scientific events and meetings, and published in English at any time.

#### Exclusion criteria

To maintain methodological rigor, the authors removed any papers that only talked about fatigue without providing specific measurements, as well as studies that tested treatments but didn’t record how common the fatigue was before the treatment began. To ensure high quality, they only used articles from professional, peer-reviewed journals, leaving out things like unpublished papers, simple presentations, or small trial tests. Studies with insufficient data were excluded, even after attempts to contact the corresponding author for clarification. Finally, in cases of uncertainty regarding eligibility, 3 additional reviewers (M.A.T., T.G.A., and C.A.W.) were consulted to reach a final consensus.

#### Data extraction

All relevant data were carefully extracted and organized using a pre-structured format developed in Microsoft Excel. Following the final selection of eligible articles, data were independently extracted by B.T., T.T.T., and Y.A.W. A standardized data extraction form was used to capture crucial information, including the first author’s name, publication year, study country, study design, study setting, interaction database, total patient count, and the number of patients who experienced cancer-related fatigue. Furthermore, details such as lower and upper confidence intervals, measures of effect (odds ratios, or ORs), the primary outcome of interest (prevalence of cancer-related fatigue), and its associated factors were also extracted. Any disagreements that arose during the data extraction process were thoroughly discussed and resolved among the authors. To enhance data accuracy and reliability, the data underwent a double-extraction process by other authors. Kappa statistics were then employed to quantify the agreement between observed and anticipated author agreements. Furthermore, a sensitivity analysis was performed to assess the robustness and reliability of the meta-analytic results.

#### Outcome measurements

The primary objective of the current systematic review and meta-analysis was to assess the pooled prevalence of cancer-related fatigue and factors associated with cancer-related fatigue among people living with cancer.

#### Quality assessment

The methodological rigor and quality of each original article were independently assessed using the Newcastle-Ottawa Scale (NOS). This comprehensive tool evaluates 3 critical domains: the quality of the statistical analysis, the robustness of the study’s methodology, and the comparability of the included articles. Each author individually applied the NOS to appraise the features of the original research. For inclusion in this study, publications with a NOS score of 6 or higher (out of a maximum score of 10) were classified as having either medium or high quality. Any discrepancies among the authors regarding the quality scores of included studies were amicably resolved through consensus and averaging. The entire quality assessment process was finalized on December 20, 2024 (Supplementary Table S1).

#### Statistical procedure

Data extraction was performed using Microsoft Excel, after which all analyses were conducted using STATA 14.0. The main characteristics of the included studies were summarized and presented through forest plots, charts, and texts. For each primary study, the standard error of prevalence was calculated based on the binomial distribution. The *Q* and *I*^2^ tests were then employed to rigorously assess the heterogeneity across the prevalence figures of the original research. The Der Simonian and Laird random-effects meta-analysis method was specifically chosen to estimate the pooled effect of cancer-related fatigue. To investigate potential sources of heterogeneity within the pooled meta-analysis of cancer-related fatigue prevalence in Ethiopia, a “leave-one-out” sensitivity analysis was also executed. Publication bias was meticulously evaluated using both Egger’s correlation and Begg’s regression intercept tests, with a significance level set at 5%. If publication bias was detected, the non-parametric trim-and-fill method was applied. Furthermore, subgroup analysis was conducted to identify factors influencing the prediction of the pooled prevalence of cancer-related fatigue within specific groups. In instances where heterogeneity was identified during the analysis, the results from the leave-one-out sensitivity analysis were instrumental in pinpointing its probable causes and understanding how the removal of a single study might impact the overall findings and the anticipated pooled prevalence.

## Results

### Search Results

**Figure 1.**
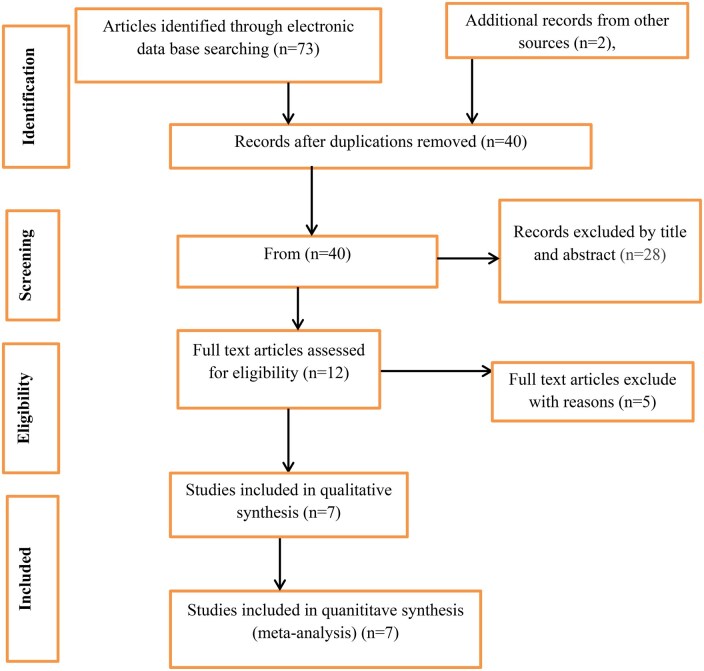
PRISMA flow diagram.

Numerous electronic search methods, including Scopus, African-wider, PsycINFO, EMBASE, Google Scholar, Psychiatry Online, World Health Organization (WHO) publications, PubMed/MEDLINE, and African Journal Online, were used to find 75 studies for this study. Redundancy led to the removal of 35 of these researchers. Furthermore, after reviewing the abstracts and titles, we excluded 28 studies because their complete texts were not available, they were not conducted in Ethiopia, their study sites and demographics varied, and they had no bearing on our evaluation. After another 12 full-text studies were assessed for eligibility according to the inclusion criteria, 5 investigations were removed for various reasons. Finally, 7 articles that met the eligibility requirements were included in this systematic review and meta-analysis (Figure 1).

### Characteristics of Included Studies

The current systematic review and meta-analysis comprised 7 primary studies on the prevalence of cancer-related fatigue and the factors associated with it among Ethiopian cancer patients. Out of all the included studies, 3 were carried out between January 20, 2018 and June 2022, while the remaining 4 were carried out between February 1, 2019 and May 2023, with publications occurring between 2019 and 2024. The cross-sectional studies included in these investigations were conducted in 3 different regions of Ethiopia. In the Amhara region, 3 articles were written,^18^^,^^27^^,^^28^ while the remaining 4 studies, 3 of which were from Addis Ababa,^19^^,^^29^ and Hawassa,^30^ respectively. A total of 2047 study respondents participated, with sample sizes ranging from a minimum 202 and maximum of 383 conducted in Addis Ababa, respectively. On the subject of measurement tool, 6 studies investigated in Amhara, Addis Ababa, and Hawassa used the brief fatigue inventory assessment tool, whereas the remaining one study, which was conducted in the Amhara region, used the multidimensional fatigue inventory −20 scale measurement tools. All original studies included in the current review were completed using a cross-sectional study design. As stated from included studies, cancer patients in the Amhara region oncology unit, 63.93%, and the University of Gondar Hospital, Ethiopia; 78.4%, had the minimum and maximum magnitude ofcancer-relatedd fatigue among patients with cancer in Ethiopi,a respectively (Table 1).

**Table 1.** Characteristics of studies included in this systematic review and meta-analysis of cancer-related fatigue among adult patients living with cancer in Ethiopia

Authors	Publication year	Region	Sample size	Prevalence of CRF (%)
Asefa et al	2024	Hawassa	297	77.4
Animaw et al	2019	Amhara	305	77.3
Nugusse et al	2017	Addis Ababa	278	74.8
Zeleke et al	2020	Amhara	326	63.9
Kassa et al	2014	Addis Ababa	383	72.3
Wondie and Hinz	2022	Amhara	256	66.7
Gebremariam et al	2018	Addis Ababa	202	78.4

### Pooled Prevalence of Cancer Related Fatigue Among Patients Living With Cancer in Ethiopia

**Figure 2.**
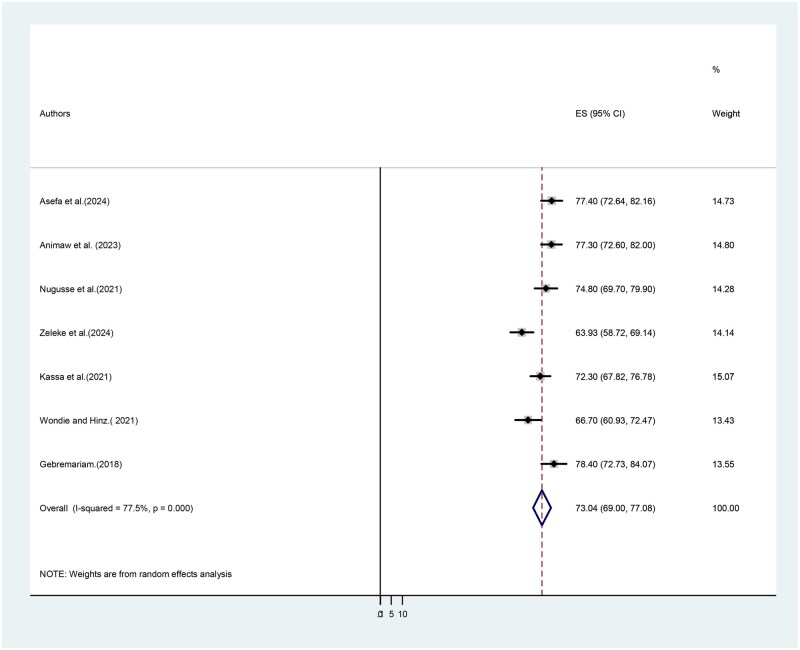
Forest plot showing the pooled prevalence of cancer-related fatigue among adult patients living with cancer in Ethiopia. The *X*-axis shows the effect size or prevalence estimates across different studies. The *Y*-axis shows the studies included in the analysis.

This systematic review and meta-analysis, drawing upon 7 published studies, methodically designed the pooled prevalence of cancer-related fatigue among individuals living with cancer in Ethiopia. The comprehensive analysis revealed a pooled prevalence of 73.04%, with a 95% confidence interval spanning from 69.00% to 77.08%. This statistically robust finding provides a crucial insight into the scale-up of the implementation of standardized screening tools at every clinical visit. Fatigue should be treated with the same clinical priority as pain within the Ethiopian context (Figure 2).

### Heterogeneity Assessment and Publication Bias

**Figure 3.**
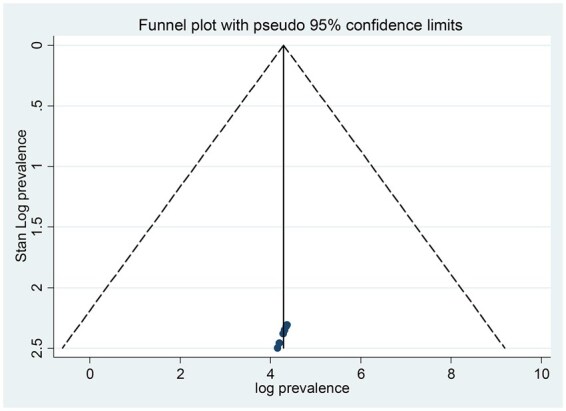
A funnel plot test meta-analysis shows an absence of publication bias. The *X*-axis indicates effect size (odds ratio) or prevalence estimates from each study included in the meta-analysis. The *Y*-axis indicates the standard error (SE) of the effect size.

In order to determine the pooled prevalence of cancer-related fatigue, a random-effects meta-analysis was employed, specifically utilizing the Der Simonian and Laird method for combining study effects. The *I*^2^ inconsistency statistic was rigorously applied to assess the heterogeneity among the included studies. An *I*^2^ value of 70% or greater was established as the threshold indicating substantial heterogeneity. To assess for publication bias, a funnel plot was created, revealing a symmetric distribution among the included studies (Figure 3). This was further supported by the Eggers test, which yielded a non-significant result (*P* = .509), confirming the absence of bias (Table 2).

**Table 2.** Egger’s test of cancer related fatigue among patients living with cancer in Ethiopia

Std_Eff	Coef.	Std. Err.	t	*P* > *t*	[95% Conf. Interval]	
Slope	90.49868	24.35615	3.72	0.094	27.8892	153.1082
Bias	−6.733613	9.47006	−0.71	0.509	−31.07718	17.60995

### Subgroup Analyses

To understand the observed variations (heterogeneity) in the prevalence of cancer-related fatigue, a detailed subgroup analysis was conducted, the results of which are presented in Table 3. The current analysis revealed significant discrepancies in the prevalence of cancer-related fatigue depending on the study periods, and regions where articles were conducted. The pooled prevalence of cancer-related fatigue among adult patients living with cancer was different when the study was conducted in different study areas, particularly because there are socio-demographic differences across the different regions of the country when studies were diverse. For example, the subgroup analysis showed that the pooled prevalence of cancer-related fatigue among adult patients living with cancer was higher in the south region (Hawassa) 77.40 (95% CI: 72.64, 82.16), followed by studies conducted in Addis Ababa 74.82 (95% CI: 71.41, 78.23). Furthermore, the pooled prevalence of cancer-related fatigue was greater in the studies carried out before 2020, with a prevalence of 73.34% (95% CI: 66.81, 79.88), than the pooled prevalence of cancer-related fatigue that were conducted after 2020, with the prevalence of 72.81% (95% CI: 66.92, 78.69).

**Table 3.** Subgroup analysis of cancer-related fatigue among adult patients living with cancer in Ethiopia

Variables	Subgroup	Number of studies	Prevalence (95% CI)	*I* ^2^ %	*P-*value
Region	Addis Ababa	3	74.82 (71.41, 78.23)	26.9	.255
	Amhara	3	69.40 (60.96, 77.84)	87.23	.000
	Hawassa	1	77.40 (72.64,82.16)	0.00	.000
Study period	<2020	3	73.34 (66.81, 79.88)	76.3	.015
	≥2020	4	72.81 (66.92, 78.69)	83.5	.000

### Sensitivity Analysis

To ensure the robustness of the pooled prevalence estimate for cancer-related fatigue, a sensitivity analysis was performed within this systematic review and meta-analysis. This analysis meticulously assessed the impact of individual studies on the overall findings, investigating heterogeneity by systematically excluding 1 author or study at a time. The results unequivocally demonstrated that the omission of any single study did not materially alter the review’s prevalence estimate. All calculated prevalence consistently remained within the anticipated 95% confidence interval, as detailed in Table 4. This confirms the stability and reliability of the overall prevalence reported.

**Table 4.** Sensitivity analysis of cancer related fatigue among adult patients living with cancer in Ethiopia

Study omitted	Estimated prevalence (%)	[95% Conf. Interval]	
Asefa et al^30^	72.282906	67.778687	76.787125
Animaw et al^28^	72.296013	67.775513	76.816513
Nugusse et al^19^	72.734108	67.9991	77.469116
Zeleke et al^18^	74.578773	71.318657	77.838882
Kassa et al (2021)^58^	73.154343	68.291542	78.017143
Wondie and Hinz^27^	74.02211	69.855202	78.189018
Gebremariam^29^	72.196846	67.828735	76.564957

### Factors Associated With Cancer Related Fatigue

**Figure 4.**
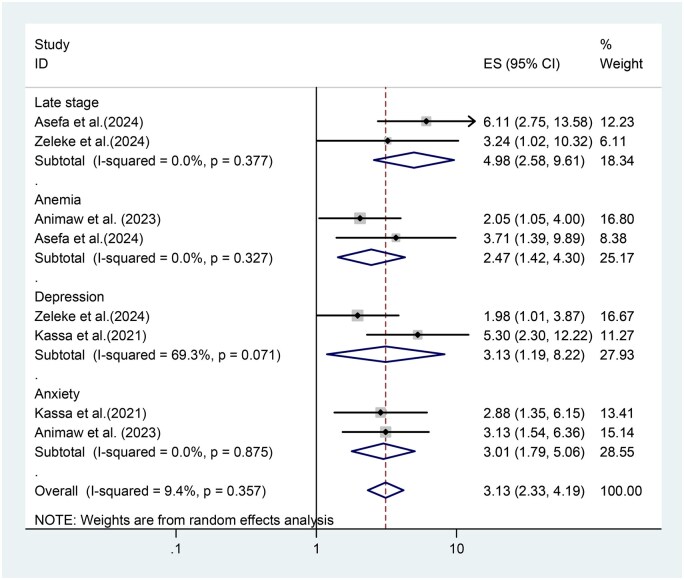
The forest plot show associated factors of cancer-related fatigue among patients living with cancer in Ethiopia.

After analysis was carried out, there are different factors associated with cancer-related fatigue among adult patients living with cancer. Late stage presentation of cancer, anemia, depression, and anxiety were associated with cancer-related fatigue among adult patients living with cancer in Ethiopia. Late stage presentations of cancer were nearly 5 times more likely to develop cancer related fatigue as compared to early stage presentation (AOR = 4.98, 95% CI: 2.58, 9.61), anemic adult patients living with cancer were nearly 2.5 times more likely to develop cancer related fatigue as compared to their complement (AOR = 2.47, 95% CI: 1.42, 4.30), depressed adult patients living with cancer were nearly 3 times more likely to develop cancer related fatigue as compared to their counterpart (AOR = 3.13, 95% CI: 1.19, 8.22), anxiety adult patients living with cancer were nearly 3 times more likely to develop cancer related fatigue as compared to their corresponding (AOR = 3.01, 95% CI: 1.79,5.06) (Figure 4).

## Discussion

The current study conducted a systematic review and meta‐analysis, estimating the Ethiopian level of pooled prevalence of cancer-related fatigue among adult cancer patients, showing an interesting result. Seven studies, with 2047 adult cancer patients, investigated across 3 region of the country, were encompassed in the current review. In general, the review findings indicated that the overall prevalence of cancer-related fatigue among adult cancer patients in Ethiopia was found to be 73.04% with a 95% CI (69.00-77.08). The pooled prevalence of cancer-related fatigue found in the current systematic review and meta-analysis was consistent with related studies conducted in the Republic of Korea and Canada. According to the previous conducted studies, the overall pooled prevalence of cancer-related fatigue among patients living with cancer was comparable with our findings 70.7%^16^ and 73%,^31^ respectively. The possible rationalization for this similarity might be that cancer-related fatigue is a common and complex symptom experienced by many cancer patients across different countries and cultures.^32^^,^^33^ The underlying mechanisms contributing to fatigue may be similar regardless of location. Moreover, it could be due to; while cultural differences might exist in how fatigue is perceived and reported, the overall experience of fatigue as a distressing and persistent symptom could be similar across cultures.^34^

With regard to the overall pooled prevalence of cancer-related fatigue patients living with cancer, to get existing literature is challenging because published articles were limited or specific/single findings. However, the pooled prevalence of cancer-related fatigue in this systematic review and meta-analysis was higher than studies conducted in China 52%,^15^ London 60.5%,^35^ Europe 49%,^13^ United Kingdom 51%,^36^ Japan 42%, and the USA 45%.^37^ The possible justification for this difference might be due to different studies using different questionnaires or scales to measure fatigue. Some tools might be more sensitive or capture different aspects of fatigue, leading to variations in reported prevalence.^38^^,^^39^ Additionally, it may be due to cultural differences in how fatigue is perceived, experienced, and reported, which could contribute. Patients in some cultures might be more or less likely to report fatigue, even if they experience it similarly.^40^^,^^41^ Furthermore, Fatigue levels can fluctuate throughout the cancer journey. If the comparison studies assessed fatigue at different time points (eg, immediately post-treatment vs. months later), it could explain some of the variation.^42^

Additionally, the current systematic review and meta-analysis identified factors associated with cancer-related fatigue among adult patients living with cancer. The pooled effect of associated factors showed that late stage presentation of cancer, anemic adult cancer patients, and adult cancer patients with depression and anxiety were significantly associated with cancer related fatigue among adult patients living with cancer.

Regarding the associated factors of cancer-related fatigue among adult patients living with cancer, late stage presentation of cancer was nearly 5 times a significant predictor of cancer-related fatigue among adult patients living with cancer. The result of this study was supported by other studies conducted in China.^43^ The possible explanation of this could be due to advanced cancers often having a larger tumor burden, meaning the cancer is more widespread in the body. Chronic systemic inflammation is the main cause of cancer-related fatigue, where a continuous state of sickness behavior in the brain is triggered by the continuous release of pro-inflammatory cytokines. HPA axis blunting, in which the body is unable to efficiently mobilize energy due to disturbed cortisol rhythms, frequently coexists with immunological dysregulation. Chemotherapy and radiation cause mitochondrial malfunction at the cellular level, harming the cell’s power plants and lowering the body’s main energy currency, adenosine triphosphate. Moreover, inflammation triggers the kynurenine pathway, which diverts tryptophan from the synthesis of serotonin to neurotoxic metabolites. This explains why physical tiredness, cognitive brain fog, and depressive symptoms frequently coexist.

On the subject of this study found that anemic adult cancer patients were nearly 2.5 times more likely to develop cancer-related fatigue as compared to their counterparts. The findings of this study were similar to studies conducted in India.^44^ The possible rationale for this might be due to anemia, a condition where the body lacks enough healthy red blood cells to carry adequate oxygen to the body’s tissues, which is a common complication of cancer and cancer treatment. It can significantly contribute to cancer-related fatigue (CRF) for several reasons.^45^^,^^46^ Furthermore, red blood cells contain hemoglobin, a protein that carries oxygen throughout the body. Anemia leads to a decrease in hemoglobin levels, resulting in reduced oxygen delivery to tissues and organs. This can cause fatigue, weakness, and shortness of breath.^47^ Generally, oxygen is essential for cellular respiration, the process by which cells convert nutrients into energy. When oxygen delivery is compromised due to anemia, energy production is impaired, leading to fatigue.^48^^,^^49^

According to this study, depressed adult patients living with cancer were nearly 3 times more likely to develop cancer-related fatigue as compared to their counterparts. The result of this study was congruent with studies conducted in Norway,^50^ India,^44^ and China.^13^ The possible rationalization for this might be that depression is associated with imbalances in neurotransmitters like serotonin, dopamine, and norepinephrine, which play a role in energy levels, motivation, and mood regulation. These imbalances can contribute to fatigue.^51^ It may also be due to depression, which can affect the hypothalamic-pituitary-adrenal (HPA) axis, leading to deregulation of cortisol, a stress hormone. Chronic stress and HPA axis dysfunction can contribute to fatigue or depression, which can decrease motivation and interest in activities, leading to decreased physical activity and subsequent fatigue. Sometimes depression can lead to changes in appetite and dietary habits, potentially resulting in nutrient deficiencies and fatigue.^52^

The current systematic review and meta-analysis found that adult patients living with cancer were nearly 3 times more likely to develop cancer-related fatigue as compared to their counterparts. The finding of this result was in line with a study conducted in Norway,^50^ India,^44^ and China.^13^ The possible justification for this may be due to the fact that both anxiety and cancer can trigger chronic inflammation in the body. This inflammation is thought to be a key factor in the development of CRF. Chronic anxiety can affect the hypothalamic-pituitary-adrenal (HPA) axis, leading to deregulation of cortisol, the stress hormone. Prolonged exposure to high cortisol levels can lead to fatigue and other health issues. Likewise, cancer diagnosis and treatment can be incredibly stressful, and anxiety amplifies these feelings.^53^^,^^54^ Constant worry and rumination can drain mental and physical energy, contributing to fatigue. Anxiety often leads to difficulty falling asleep, staying asleep, or experiencing restful sleep. Poor sleep quality can significantly worsen fatigue.^55^^,^^56^ Occasionally, anxiety can interfere with a person’s ability to cope with the challenges of cancer treatment and its side effects, making them feel more overwhelmed and exhausted. Anxiety can lead to decreased motivation and interest in physical activity. This can result in muscle weakness and fatigue over time.^57^

### Limitations of the Study

Even though this systematic review and meta-analysis provide numerous advantages, the combined effect of cancer-related fatigue among adult patients living with cancer has the following limitation: The meta-analysis’s conclusions might not apply to all cancer patients because the included studies might have concentrated on particular cancer types or patient demographics. Data availability may restrict the meta-analysis, especially in certain demographics or geographical areas. For instance, there may not be many studies on weariness linked to cancer in some parts of Ethiopia. Furthermore, confounding factors such as high rates of anemia, malnutrition, and financial stress, which are common among Ethiopian cancer patients, make it difficult to determine if the fatigue is strictly cancer-related or a result of these overlapping life challenges. Finally, a lack of longitudinal data means that while we know many patients suffer from fatigue during treatment, we have very little evidence regarding the long-term persistence of these symptoms among Ethiopian cancer survivors.

## Conclusion and Recommendation

The current systematic review and meta-analysis reported that the pooled prevalence of cancer-related fatigue among adult patients living with cancer in Ethiopia was high. Regarding the associated factors, the pooled effect of associated factors showed that late stage presentation of cancer, anemic cancer patient, depression, and anxiety cancer patients were significantly associated with cancer-related fatigue among adult patients living with cancer. It’s better, health care providers in oncology treatment facilities should routinely screen cancer patients for both psychological determinants and fatigue, and offer integrated interventions that address both simultaneously. Policies should be implemented to ensure access to mental health services for cancer patients, including screening and treatment for anemia. Finally, for future researchers, it is better to conduct another study design, specifically a randomized controlled trial.

## Supplementary Material

vdag105_Supplementary_Data

## Data Availability

All relevant data are available within the manuscript.

## References

[1] Bray F , Ferlay J, Soerjomataram I, Siegel RL, Torre LA, Jemal A. Global cancer statistics 2018: GLOBOCAN estimates of incidence and mortality worldwide for 36 cancers in 185 countries. CA Cancer J Clin. 2018;68:394-424. 10.3322/caac.21492 30207593

[2] Parkin DM , Bray F, Ferlay J, Jemal A. Cancer in Africa 2012. Cancer Epidemiol Biomarkers Prev. 2014;23:953-966. 10.1158/1055-9965.EPI-14-0281 24700176

[3] Soerjomataram I , Bray F. Planning for tomorrow: global cancer incidence and the role of prevention 2020-2070. Nat Rev Clin Oncol. 2021;18:663-672. 10.1038/s41571-021-00514-z 34079102

[4] Mathers CD , Loncar D. Projections of global mortality and burden of disease from 2002 to 2030. PLoS Med. 2006;3:e442. 10.1371/journal.pmed.0030442 17132052 PMC1664601

[5] Hampel H. NCCN increases the emphasis on genetic/familial high-risk assessment in colorectal cancer. J Natl Compr Canc Netw. 2014;12:829-831. 10.6004/jnccn.2014.0200 24853227

[6] Woldu M , Legese D, Abamecha F, Berha A. The prevalence of cancer and its associated risk factors among patients visiting oncology unit, Tikur Anbessa Specialized Hospital, Addis Ababa-Ethiopia. J Cancer Sci Ther. 2017;9:11.

[7] Sprod LK , Fernandez ID, Janelsins MC, et al Effects of yoga on cancer-related fatigue and global side-effect burden in older cancer survivors. J Geriatr Oncol. 2015;6:8-14. 10.1016/j.jgo.2014.09.184 25449185 PMC4297736

[8] Wang XS , Woodruff JF. Cancer-related and treatment-related fatigue. Gynecol Oncol. 2015;136:446-452. 10.1016/j.ygyno.2014.10.013 25458588 PMC4355326

[9] Hofman M , Ryan JL, Figueroa-Moseley CD, Jean-Pierre P, Morrow GR. Cancer-related fatigue: the scale of the problem. Oncologist. 2007;12:4-10. 10.1634/theoncologist.12-S1-4 17573451

[10] Bower JE. Cancer-related fatigue—mechanisms, risk factors, and treatments. Nat Rev Clin Oncol. 2014;11:597-609. 10.1038/nrclinonc.2014.127 25113839 PMC4664449

[11] Morrow GR. Cancer‐related fatigue: causes, consequences, and management. Oncologist. 2007;12:1-3. 10.1634/theoncologist.12-S1-1 17573450

[12] Campos M , Hassan B, Riechelmann R, Del Giglio A. Cancer-related fatigue: a practical review. Ann Oncol. 2011;22:1273-1279. 10.1093/annonc/mdq458 21325448

[13] Al Maqbali M , Al Sinani M, Al Naamani Z, Al Badi K, Tanash MI. Prevalence of fatigue in patients with cancer: a systematic review and meta-analysis. J Pain Symptom Manage. 2021;61:167-189. e14. 10.1016/j.jpainsymman.2020.07.037 32768552

[14] Abrahams H , Gielissen M, Schmits I, Verhagen C, Rovers M, Knoop H. Risk factors, prevalence, and course of severe fatigue after breast cancer treatment: a meta-analysis involving 12 327 breast cancer survivors. Ann Oncol. 2016;27:965-974. 10.1093/annonc/mdw099 26940687

[15] Ma Y , He B, Jiang M, et al Prevalence and risk factors of cancer-related fatigue: a systematic review and meta-analysis. Int J Nurs Stud. 2020;111:103707. 10.1016/j.ijnurstu.2020.103707 32920423

[16] Kang Y-E , Yoon J-H, Park N-H, Ahn Y-C, Lee E-J, Son C-G. Prevalence of cancer-related fatigue based on severity: a systematic review and meta-analysis. Sci Rep. 2023;13:12815. 10.1038/s41598-023-39046-0 37550326 PMC10406927

[17] Curt GA , Breitbart W, Cella D, et al Impact of cancer-related fatigue on the lives of patients: new findings from the fatigue coalition. Oncologist. 2000;5:353-360. 10.1634/theoncologist.5-5-353 11040270

[18] Zeleke G , Worku WZ, Ayele D. Prevalence and associated factors of cancer-related fatigue among adult patients with cancer attending oncology units: an institution-based cross-sectional study design in the Amhara region, Ethiopia, 2022. BMJ Public Health. 2024;2:e000884. 10.1136/bmjph-2023-000884 40018631 PMC11816097

[19] Nugusse T , Lemlem SB, Deressa J, Kisa S. Prevalence of fatigue and associated factors among cancer patients attending Tikur Anbessa Specialized Hospital, Addis Ababa, Ethiopia. Cancer Manag Res. 2021;13:1909-1916. 10.2147/CMAR.S291298 33658853 PMC7917325

[20] Sukartini T , Efendi F, Estiadewi PS, Anggraeni NPDA. The experiences of cancer-related fatigue among adult cancer patients: a systematic review. Jurnal Ners. 2019;14:35.

[21] Berger AM , Mooney K, Alvarez-Perez A, et al; National Comprehensive Cancer Network. Cancer-related fatigue, version 2.2015. J Natl Comprehen Cancer Netw. 2015;13:1012-1039. 10.6004/jnccn.2015.0122 PMC5499710 26285247

[22] Ross DD , Alexander CS. Management of common symptoms in terminally ill patients: part I. fatigue, anorexia, cachexia, nausea and vomiting. Am Fam Physician. 2001;64:807-814. 11563572

[23] Mitchell SA , Beck SL, Hood LE, Moore K, Tanner ER. Putting evidence into practice: evidence-based interventions for fatigue during and following cancer and its treatment. Clin J Oncol Nurs. 2007;11:99-113. 10.1188/07.CJON.99-113 17441401

[24] Lim E-J , Ahn Y-C, Jang E-S, Lee S-W, Lee S-H, Son C-G. Systematic review and meta-analysis of the prevalence of chronic fatigue syndrome/myalgic encephalomyelitis (CFS/ME). J Transl Med. 2020;18:100-115. 10.1186/s12967-020-02269-0 32093722 PMC7038594

[25] Mitchell SA , Hoffman AJ, Clark JC, et al Putting evidence into practice: an update of evidence-based interventions for cancer-related fatigue during and following treatment. Clin J Oncol Nurs. 2014;18:38-58. 10.1188/14.CJON.S3.38-58 25427608

[26] Page MJ , McKenzie JE, Bossuyt PM, et al The PRISMA 2020 statement: an updated guideline for reporting systematic reviews. BMJ. 2021;372:n71. 10.1136/bmj.n71 33782057 PMC8005924

[27] Wondie Y , Hinz A. Application of the multidimensional fatigue inventory to Ethiopian cancer patients. Front Psychol. 2021;12:687994. 10.3389/fpsyg.2021.687994 34925119 PMC8674181

[28] Animaw L , Woldegiorgis Abate T, Endeshaw D, Tsegaye D. Fatigue and associated factors among adult cancer patients receiving cancer treatment at oncology unit in Amhara region, Ethiopia. PLoS One. 2023;18:e0279628. 10.1371/journal.pone.0279628 36607977 PMC9821493

[29] Gebremariam GT , Anshabo AT, Tigeneh W, Engidawork E. Validation of the amharic version of the brief fatigue inventory for assessment of cancer-related fatigue in Ethiopian cancer patients. J Pain Symptom Manage. 2018;56:264-272. 10.1016/j.jpainsymman.2018.04.015 29753101

[30] Asefa T , Bitew G, Tezera H, Tesfaye W. Prevalence of cancer-related fatigue, associated factors and adult cancer patients’ experiences at Hawassa University Comprehensive Specialized Hospital in Ethiopia: a mixed methods study. Front Oncol. 2024;14:1480246. 10.3389/fonc.2024.1480246 39555447 PMC11563971

[31] Oberoi S , Huang B, Rabbani R, et al Prevalence and factors associated with cancer‐related fatigue among children and adolescents undergoing cancer treatment: a systematic review and meta‐analysis. Cancer Med. 2024;13:e70502. 10.1002/cam4.70502 39660372 PMC11632266

[32] Weis J. Cancer-related fatigue: prevalence, assessment and treatment strategies. Expert Rev Pharmacoecon Outcomes Res. 2011;11:441-446. 10.1586/erp.11.44 21831025

[33] Al Maqbali M. Cancer-related fatigue: an overview. Br J Nurs. 2021;30:S36-S43. 10.12968/bjon.2021.30.4.S36 33641391

[34] Whitehead LC , Unahi K, Burrell B, Crowe MT. The experience of fatigue across long-term conditions: a qualitative meta-synthesis. J Pain Symptom Manage. 2016;52:131-143.e1. 10.1016/j.jpainsymman.2016.02.013 27233142

[35] Minton O , Richardson A, Sharpe M, Hotopf M, Stone P. A systematic review and meta-analysis of the pharmacological treatment of cancer-related fatigue. J Natl Cancer Inst. 2008;100:1155-1166. 10.1093/jnci/djn250 18695134

[36] Prue G , Rankin J, Allen J, Gracey J, Cramp F. Cancer-related fatigue: a critical appraisal. Eur J Cancer. 2006;42:846-863. 10.1016/j.ejca.2005.11.026 16460928

[37] Wang XS , Zhao F, Fisch MJ, et al Prevalence and characteristics of moderate to severe fatigue: a multicenter study in cancer patients and survivors. Cancer. 2014;120:425-432. 10.1002/cncr.28434 24436136 PMC3949157

[38] Kunasegaran K , Ismail AMH, Ramasamy S, Gnanou JV, Caszo BA, Chen PL. Understanding mental fatigue and its detection: a comparative analysis of assessments and tools. PeerJ. 2023;11:e15744. 10.7717/peerj.15744 37637168 PMC10460155

[39] Jason LA , Evans M, Brown M, et al Fatigue scales and chronic fatigue syndrome: issues of sensitivity and specificity. Disability studies quarterly: DSQ. 2011;31(1):1375. 10.18061/dsq.v31i1.1375. PMC3181109 21966179

[40] Steege LM , Rainbow JG. Fatigue in hospital nurses—‘supernurse’culture is a barrier to addressing problems: a qualitative interview study. Int J Nurs Stud. 2017;67:20-28. 10.1016/j.ijnurstu.2016.11.014 27894030

[41] Minton O , Berger A, Barsevick A, et al Cancer‐related fatigue and its impact on functioning. Cancer. 2013;119:2124-2130. 10.1002/cncr.28058 23695924

[42] Berger AM , Mitchell SA, Jacobsen PB, Pirl WF. Screening, evaluation, and management of cancer‐related fatigue: ready for implementation to practice? CA Cancer J Clin. 2015;65:190-211. 10.3322/caac.21268 25760293

[43] Huang S-T , Ke X, Yu X-Y, Wu Y-X, Huang Y-X, Liu D. Risk factors for cancer-related fatigue in patients with colorectal cancer: a systematic review and meta-analysis. Support Care Cancer. 2022;30:10311-10322. 10.1007/s00520-022-07432-5 36318342

[44] DSilva F , Singh P, Javeth A. Determinants of cancer-related fatigue among cancer patients: a systematic review. J Palliat Care. 2023;38:432-455. 10.1177/08258597221131133 36245333

[45] Hoque E , Karim S, Hoque M, Hoque AN, Elahi I. Management of anemia in cancer patients. Anwer Khan Mod Med Coll J. 2020;11:66-72. 10.3329/akmmcj.v11i1.45670

[46] Nasrullah IR , Khalid N. Anemia symptoms, causes, prevention, diagnosis and treatment. J Biomed Engg Res. 2019;1(1).

[47] Obeagu EI , Ali MM, Alum EU, Obeagu GU, Ugwu PO, Bunu UO. An update of aneamia in adults with heart failure. 2023;11(2):1–16.

[48] Tardy A-L , Pouteau E, Marquez D, Yilmaz C, Scholey A. Vitamins and minerals for energy, fatigue and cognition: a narrative review of the biochemical and clinical evidence. Nutrients. 2020;12:228. 10.3390/nu12010228 31963141 PMC7019700

[49] Musallam KM , Taher AT. Iron deficiency beyond erythropoiesis: should we be concerned? Curr Med Res Opin. 2018;34:81-93. 10.1080/03007995.2017.1394833 29050512

[50] Fosså SD , Dahl AA, Loge JH. Fatigue, anxiety, and depression in long-term survivors of testicular cancer. J Clin Oncol. 2003;21:1249-1254. 12663711 10.1200/JCO.2003.08.163

[51] Zielinski MR , Systrom DM, Rose NR. Fatigue, sleep, and autoimmune and related disorders. Front Immunol. 2019;10:1827. 10.3389/fimmu.2019.01827 31447842 PMC6691096

[52] Lai FH-Y , Uscinska M, Yan EW-H. Hypothalamic-pituitary-adrenal (HPA) axis and chronic fatigue syndrome in older adults: the rehabilitation perspectives. In: *Neuroimaging: Neurobiology, Multimodal and Network Applications*. London: IntechOpen; 2020;1:375.

[53] Horneber M , Fischer I, Dimeo F, Rüffer JU, Weis J. Cancer-related fatigue: epidemiology, pathogenesis, diagnosis, and treatment. Dtsch Arztebl Int. 2012;109:161-171.quiz 172. 10.3238/arztebl.2012.0161 22461866 PMC3314239

[54] LaVoy EC , Fagundes CP, Dantzer R. Exercise, inflammation, and fatigue in cancer survivors. Exerc Immunol Rev. 2016;22:82-93. 26853557 PMC4755327

[55] Trill MD. Anxiety and sleep disorders in cancer patients. Eur J Cancer Suppl. 2013;11:216-224. 10.1016/j.ejcsup.2013.07.009 PMC4041166 26217130

[56] Brown WJ , Wilkerson AK, Boyd SJ, Dewey D, Mesa F, Bunnell BE. A review of sleep disturbance in children and adolescents with anxiety. J Sleep Res. 2018;27:e12635. 10.1111/jsr.12635 29193443

[57] Dziubek W , Kowalska J, Kusztal M, et al The level of anxiety and depression in dialysis patients undertaking regular physical exercise training: a preliminary study. Kidney Blood Press Res. 2016;41:86-98. 10.1159/000368548 26872253

[58] Kassa S , Mengistu D, Woldemariam EB, Assefa M, Kebede MA. Prevalence and Factors Associated with Cancer Related Fatigue Among Cancer Patients Attending Tikur Anbessa Specialized Referral Hospital, Addis Ababa, Ethiopia. 2020.

