# Supplementary material for: Cancer-related fatigue among adult patients living with cancer in Ethiopia: A systematic review and meta-analysis
Source: Neurooncol Adv. 2026 Apr 17;8(1):vdag105. doi: 10.1093/noajnl/vdag105 (PMC13161574; doi:10.1093/noajnl/vdag105)
Supplement: vdag105_Supplementary_Data [file vdag105_supplementary_data.zip › Supplementary Table 1.DOCX]

Table_S1 Quality assessment of cancer related fatigue and its associated factors in Ethiopia included studies in this meta-analysis and systematic review.

| Author, year of  Publication | Q1 | Q2 | Q3 | Q4 | Q5 | Q6 | Q7 | Q8 | Q9 | Total score (9%) |
| --- | --- | --- | --- | --- | --- | --- | --- | --- | --- | --- |
| Assefa et al.(2024) | Y | Y | Y | Y | Y | Y | Y | Y | Y | 9 |
| Animaw et al. (2023) | Y | Y | Y | Y | NA | Y | Y | Y | Y | 8 |
| Nugusse et al.(2021) | Y | Y | Y | Y | Y | Y | Y | Y | Y | 9 |
| Zeleke et al.(2024) | Y | Y | Y | Y | Y | Y | Y | NA | Y | 8 |
| Kassa et al.(2021) | Y | Y | Y | Y | Y | Y | Y | Y | Y | 9 |
| Wondie and Hinz.( 2021) | Y | NA | Y | Y | Y | Y | Y | Y | Y | 8 |
| Gebremariam.(2018) | Y | Y | Y | Y | Y | Y | NR | Y | Y | 8 |

**Key:** **Y**= Yes; **NR**= Not reported, **NA**=Not appropriate

**Question codes:**

1. Was the sample frame appropriate to address the target population?

2. Were study participants sampled in an appropriate way?

3. Was the sample size adequate?

4. Were the study subjects and the setting described in detail?

5. Was the data analysis conducted with sufficient coverage of the identified sample?

6. Were valid methods used for the identification of the condition?

7. Was the condition measured in a standard, reliable way for all participants?

8. Was there appropriate statistical analysis?

9. was the response rate adequate, and if not, was the low response rate managed appropriately?
